# A systematic analysis of diagnostic performance for Alzheimer's disease using structural MRI

**DOI:** 10.1093/psyrad/kkac001

**Published:** 2022-03-09

**Authors:** Jiangping Wu, Kun Zhao, Zhuangzhuang Li, Dong Wang, Yanhui Ding, Yongbin Wei, Han Zhang, Yong Liu

**Affiliations:** School of Artificial Intelligence, Beijing University of Posts and Telecommunications, Beijing, 100876, China; Beijing Advanced Innovation Centre for Biomedical Engineering, School of Biological Science and Medical Engineering, Beihang University, Beijing, 100191, China; School of Artificial Intelligence, Beijing University of Posts and Telecommunications, Beijing, 100876, China; School of Information Science and Engineering, Shandong Normal University, Ji'nan, 250014, China; School of Information Science and Engineering, Shandong Normal University, Ji'nan, 250014, China; School of Artificial Intelligence, Beijing University of Posts and Telecommunications, Beijing, 100876, China; School of Artificial Intelligence, Beijing University of Posts and Telecommunications, Beijing, 100876, China; School of Artificial Intelligence, Beijing University of Posts and Telecommunications, Beijing, 100876, China; Center for Artificial Intelligence in Medical Imaging, Beijing University of Posts and Telecommunications, Beijing, 100876, China

**Keywords:** Alzheimer's disease, diagnosis, heterogeneity, sMRI, meta-analysis

## Abstract

**Background:**

Alzheimer's disease (AD) is one of the most common neurodegenerative disorders in the elderly. Although numerous structural magnetic resonance imaging (sMRI) studies have reported diagnostic models that could distinguish AD from normal controls (NCs) with 80–95% accuracy, limited efforts have been made regarding the clinically practical computer-aided diagnosis (CAD) system for AD.

**Objective:**

To explore the potential factors that hinder the clinical translation of the AD-related diagnostic models based on sMRI.

**Methods:**

To systematically review the diagnostic models for AD based on sMRI, we identified relevant studies published in the past 15 years on PubMed, Web of Science, Scopus, and Ovid. To evaluate the heterogeneity and publication bias among those studies, we performed subgroup analysis, meta-regression, Begg's test, and Egger's test.

**Results:**

According to our screening criterion, 101 studies were included. Our results demonstrated that high diagnostic accuracy for distinguishing AD from NC was obtained in recently published studies, accompanied by significant heterogeneity. Meta-analysis showed that many factors contributed to the heterogeneity of high diagnostic accuracy of AD using sMRI, which included but was not limited to the following aspects: (i) different datasets; (ii) different machine learning models, e.g. traditional machine learning or deep learning model; (iii) different cross-validation methods, e.g. *k*-fold cross-validation leads to higher accuracies than leave-one-out cross-validation, but both overestimate the accuracy when compared to validation in independent samples; (iv) different sample sizes; and (v) the publication times. We speculate that these complicated variables might be the adverse factor for developing a clinically applicable system for the early diagnosis of AD.

**Conclusions:**

Our findings proved that previous studies reported promising results for classifying AD from NC with different models using sMRI. However, considering the many factors hindering clinical radiology practice, there would still be a long way to go to improve.

## Introduction

Alzheimer's disease (AD) is one of the most common neurodegenerative diseases in the elderly and it accounts for 60–70% of all dementia. There are >55 million people worldwide living with dementia, and a recent report according to Alzheimer's Disease International speculated that it would reach 78 million in 2030 (Gauthier *et al*., 2021).

AD is one of the top five leading causes of death among people older than 65 years in China (Zhou *et al*., 2019), and it is usually characterized by memory impairment, aphasia, apraxia, agnosia, visuospatial deficit, executive dysfunction (2021 Alzheimer's Disease Facts and Figures, 2021). Given the limited progression of pharmacological treatments for AD, convergent evidence has suggested that an early diagnosis plays a crucial role in delaying the progress of AD (Dubois *et al*., 2016; Rasmussen & Langerman, 2019; Vaz & Silvestre, 2020). AD leads to neuronal loss and, ultimately, atrophy: first in the medial temporal lobe (especially in the bilateral hippocampi) and later in the dementia stage in widespread cortical areas (Pini *et al*., 2016; Poulakis *et al*., 2018; Whitwell *et al*., 2012). Machine learning algorithms aim to detect the atrophy patterns and robustly distinguish them from normal aging (which partly also causes atrophy in the medial temporal lobe). Structural magnetic resonance imaging (sMRI), a powerful technique for noninvasive *in vivo* imaging of the human brain, has been used successfully in investigating the patterns of brain atrophy as an estimate of regional neurodegeneration of AD (Rathore *et al*., 2020). Many studies have reported high accuracy (i.e. 80–95%) for classifying AD from normal controls (NC) based on different sMRI features (for reviews, see Chavez-Fumagalli *et al*., 2021; Rathore *et al*., 2017). Some correct ratios are even higher than 95% in some diagnostic models based on deep learning (Zhang *et al*., 2021). Therefore, it would be very likely to be clinically translated in assisting with the early diagnosis of AD; although several existing computer-aided diagnosis (CAD) tools are already commercially available (https://www.cortechslabs.com/neuroquant; https://www.cneuro.com/cmri; https://mediaire.de/product/mdbrain; https://icometrix.com/products/icobrain-dm; https://jung-diagnostics.de/de/diagnostics), they only assess the volume of specific brain regions, which needs to be further assessed by the radiologists. The reality is that a CAD system that can automatically distinguish AD from the outpatients has rarely been reported.

The primary purpose of the present study is to investigate and discuss the possible reasons for hindering the clinical translation of the AD-related diagnostic models. First, we first performed a systematic review of the relevant studies between January 2006 and September 2021. Subsequently, a meta-analysis was introduced to evaluate the heterogeneity among the included studies quantitatively. Specifically, the heterogeneity of different stratified frames, including "dataset", "machine learning model," "cross-validation method," "sample size," and "publication time" were carefully evaluated. Finally, we assessed the robustness of the heterogeneous results of the study using different methods (i.e. subgroup analysis, meta-regression, and sensitivity analysis).

## Materials and Methods

### Search strategy

According to the Preferred Reporting Items for Systematic Reviews and Meta-Analysis (PRISMA) guidelines (Liberati *et al*., 2009; Moher *et al*., 2009), we conducted a systematic literature review of previous studies that implemented sMRI to classify/predict AD from NC. To identify the original relevant articles for this paper, we adopted the following search strategy: (i) searching the titles and abstracts in English that published from January 2006 to September 2021 on PubMed, Web of Science, Scopus, and Ovid; and (ii) using Boolean operators in an advanced query: the search terms were concatenated as ("classification" OR "diagnostic" OR "predict*") AND ("MRI" OR "Magnetic Resonance Imaging") AND ("Alzheimer*"). Note that duplicate articles were removed from further analysis.

### Inclusion criteria

To restrict studies for our aim, we performed the peer-reviewed research strategy to screen all retrieved articles (Sarica *et al*., 2017; Song *et al*., 2020). In particular, two examiners (J.W. and Z.L.) independently evaluated the full manuscripts using the following criteria: (i) focused on the classification of Alzheimer's disease and established diagnostic models based on sMRI; (ii) total sample size was larger than 20; and (iii) reported the true positive (TP), false positive (FP), true negative (TN), and false negative (FN) value or these measures could be inferred. If J.W. and Z.L. had a conflict of understanding, the third examiner (Y.L.) would be the arbiter to resolve their conflicts. If more than one diagnostic model was reported in one study, we would only retain the model with the highest accuracy.

### Investigations of heterogeneity

To estimate the performance of the diagnostic models, we first calculated the diagnostic odds ratio (DOR) for each model. DOR is defined as (TP × TN)/(FP × FN) and is used to measure the effectiveness of a diagnostic test (Glas *et al*., 2003). Then univariate meta-analysis was performed for DOR with R package "meta" (https://cran.r-project.org/web/packages/meta/, v.5.0–1) to estimate the heterogeneity and publication bias among studies. The confidence level for all calculations was set to 95% (95% CI) (Chavez-Fumagalli *et al*., 2021; Song *et al*., 2020). The *I*^2^ was used to reflect the proportion of heterogeneity in the total variation of effect size. The definition of *I*^2^ is (Huedo-Medina *et al*., 2006):

\begin{eqnarray*}
{I^2} &=& \left \{ \begin{array}{ll} \frac{Q - d}{Q} \times 100\% & \quad Q > \left(k - 1\right)\\
0 & \quad Q \le \left(k - 1\right) \end{array}\right.\\
Q &=& \mathop \sum \limits_{i = 1}^k {W_i}\left( {{Y_i} - M} \right)
\end{eqnarray*}

where *W_i_, Y_i_*, and *M* represent weight for the study *i*, the effect size for the study *i*, and the summary effect, respectively. *i* is the *i*th study, *k* the total number of studies included, and *d* the degree of freedom of *Q*. The calculation of *I*^2^ is based on the random-effect model (Barili *et al*., 2018; Clark & Linzer, 2014).

A significantly heterogeneous result would be reported if *I*^2^ > 50% and *P* < 0.1 (Higgins & Thompson, 2002; Huedo-Medina *et al*., 2006). To further explore which factor influenced the heterogeneity of diagnostic test accuracy, we performed heterogeneity analyses for DOR with different stratified frames (i.e. "dataset," "machine learning model," "cross-validation method," "sample size," and "publication time"). In addition, meta-regression analysis can explore the factors and quantitatively describe the heterogeneity of different variables in meta-analysis.

### Assessment of reporting bias

Reporting bias is known as publication bias, which has an enormous impact on the effectiveness of a systematic review and its meta-analysis (Dwan *et al*., 2013). It occurs when studies with favorable results are more likely to be published than studies with unfavorable results (van Enst *et al*., 2014). Generally, the most common method to discriminate publication bias is to draw a funnel plot and then evaluate publication bias by examining the asymmetry of the funnel plot (Harbord *et al*., 2006). The trim-and-fill method was used to describe the asymmetry of the funnel plot (Duval & Tweedie, 2000). Begg's test and Egger's test were introduced to quantitatively assess publication bias via funnel plot asymmetry (Leeflang, 2014; van Enst *et al*., 2014).

## Results

**Figure 1:**
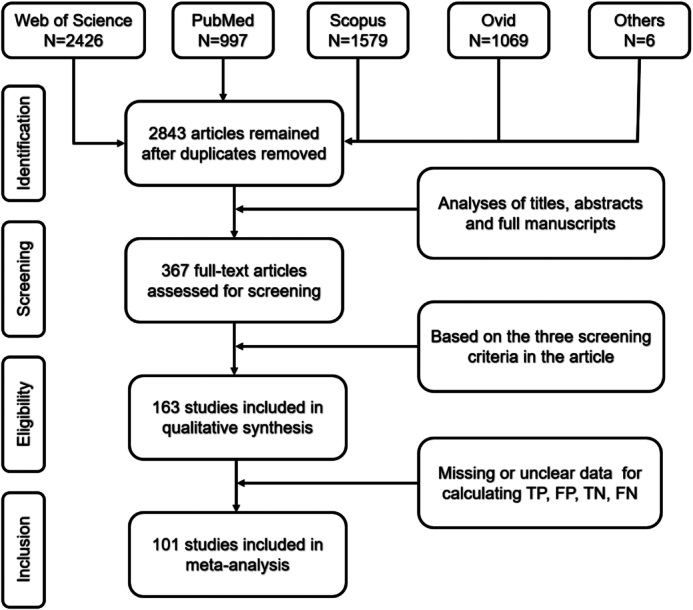
The workflow diagram of PRISMA in the study.

A total of 2837 nonduplicate articles were identified. During the screening process, 367 articles were retained through our initial examination of their titles and abstracts. Then, examiners double-checked and excluded 204 articles according to the previously mentioned criteria. Of the remaining 163 papers, the TP, FP, FN, and FN were reported or correctly calculated in 101 articles. Note that some articles may contain multiple validation results. Herein, 116 records were finally included in our meta-analysis (Fig. 1).

### Preliminary analysis of the diagnostic model

**Figure 2:**
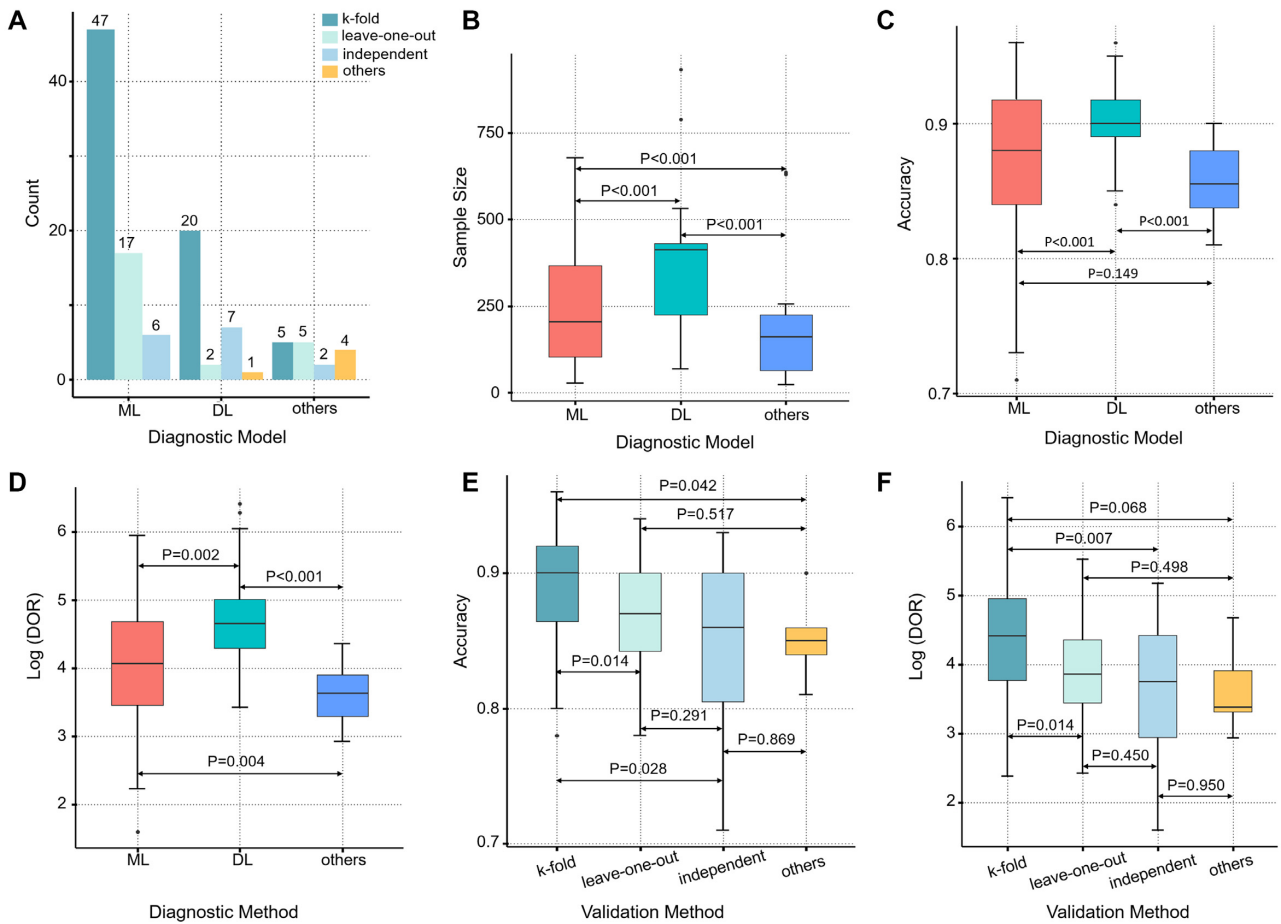
(A) The number of different validation methods in different diagnostic models. (B) The differences in the sample size of different diagnostic models. (C) The differences in diagnostic accuracy of different models. (D) The differences in diagnostic accuracy of different validation methods. (E) The differences in diagnostic odds ratio of different models. (F) The differences in diagnostic odds ratio of different validation methods.

First, we conducted a preliminary exploration of the AD diagnosis models based on sMRI. Among the included papers, the number of studies of three types of diagnostic model [i.e. machine learning (ML), deep learning (DL), and others] was significantly different (60, 26, and 14%, respectively; Fig. 2a). The sample sizes of ML, DL, and other models were significantly different and the mean sample size in DL was the largest (Fig. 2b). The accuracy of DL models was markedly higher than the other two types of model (all *P* < 0.001). In contrast, there was no significant difference between ML models and models of others (*P* = 0.149) (Fig. 2c). However, for DOR, there were substantial differences among the three types of diagnostic model (all *P* < 0.005) (Fig. 2d). In addition, the cross-validation methods can be primarily divided into four categories (*k*-fold, leave-one-out, independent, and others), where the *k*-fold cross-validation method was the most commonly used and with the highest accuracy and DOR (Fig. 2e and f).

**Figure 3:**
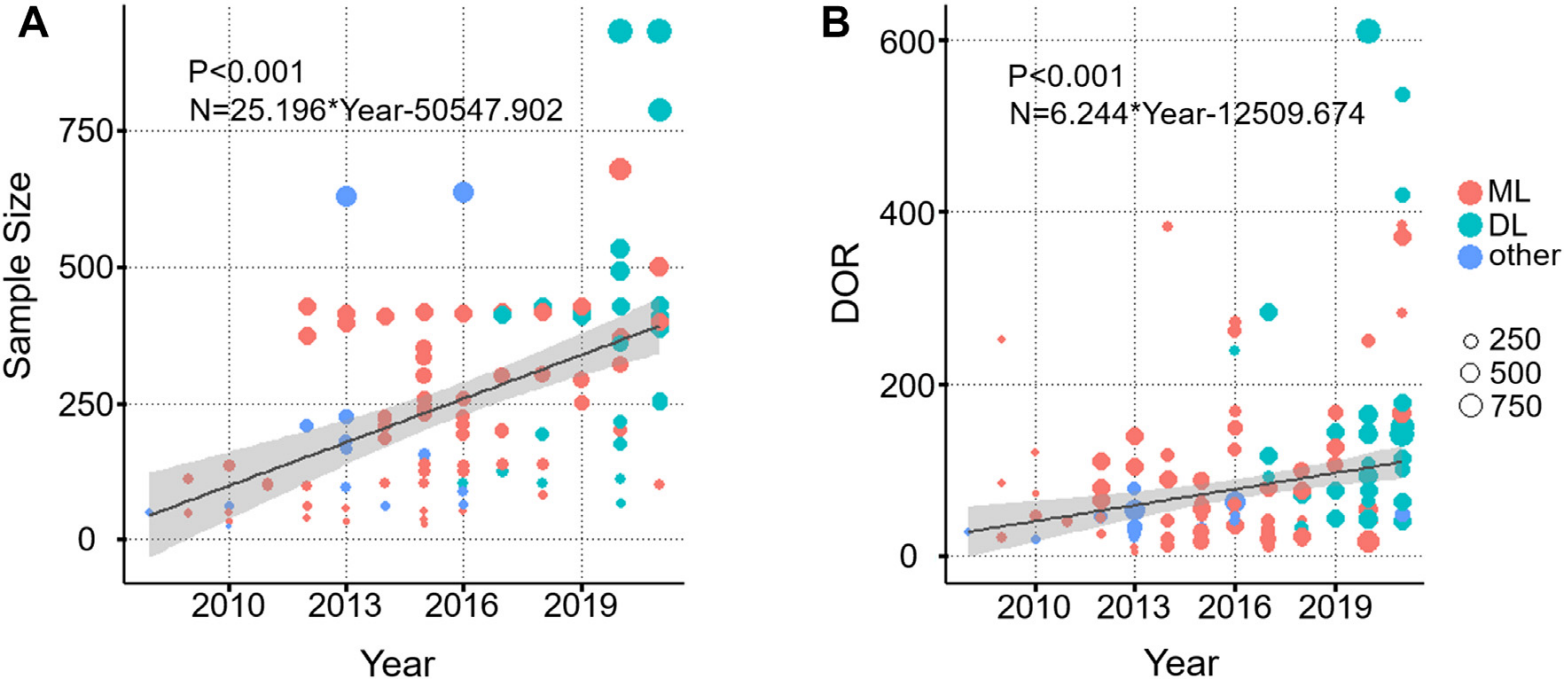
(A) The sample size of AD diagnostic model changes with the year of publication. (B) The trend of the DOR of a diagnostic model for AD with the year of publication. The different colors and sizes of the circles correspond to different models for AD diagnosis and sample sizes of diagnostic models.

As shown in Fig. 3, the study sample size significantly increased in recent years (*P* < 0.001). Similarly, the diagnostic accuracy also displayed a significant increasing trend along with the year of publication (*P* < 0.001).

### Results of assessment for heterogeneity

**Figure 4:**
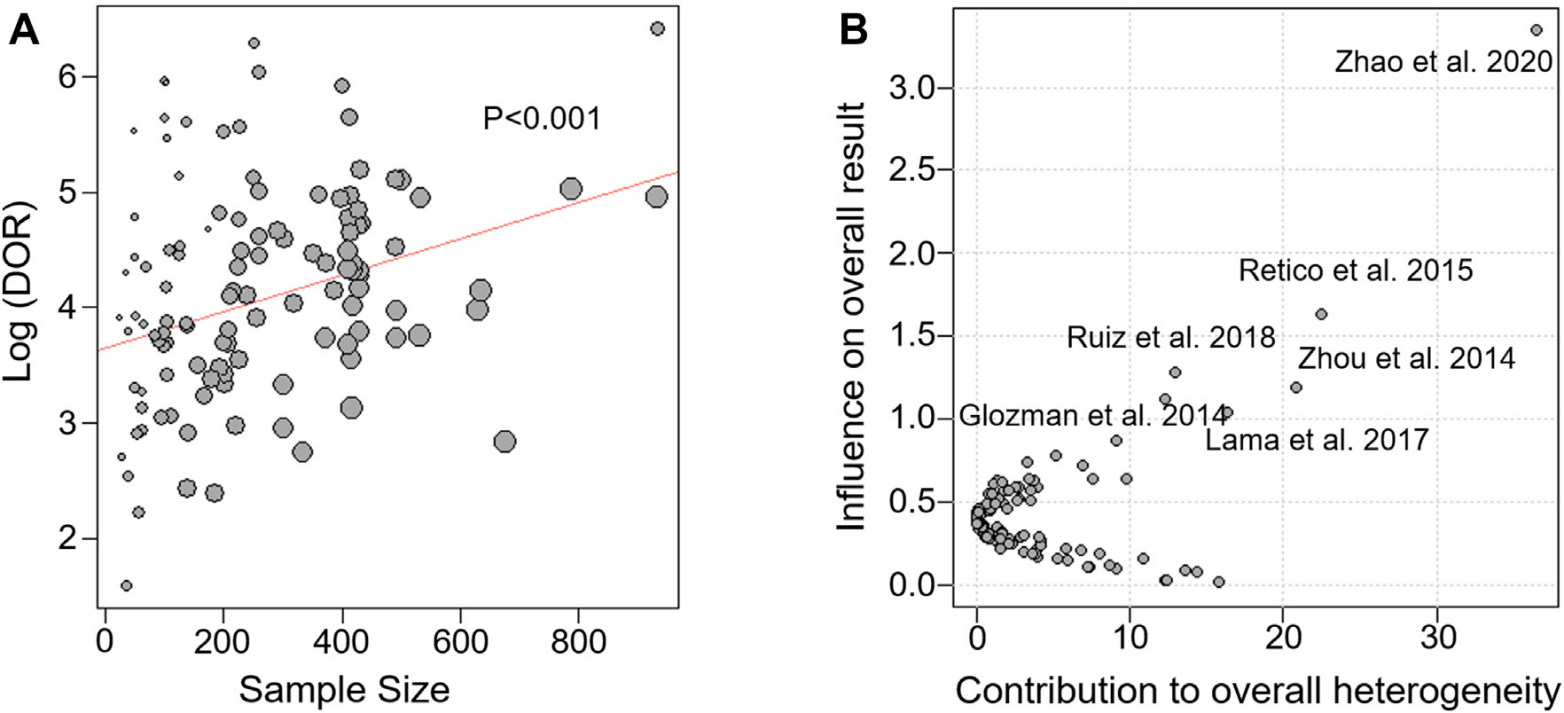
(A) Bubble plot of sample size in meta-regression. Different circles correspond to different studies. The size of the circle is inversely proportional to the variance of the estimated effect value. (B) A Baujat plot explores the heterogeneity in a meta-analysis. Different circles also correspond to different studies.

As shown in Table 1, the value of *I*^2^ was 73% (*P* < 0.001), indicating high heterogeneity among the included studies. To explore the reasons for the heterogeneity, we then compared different subgroups according to the stratification from five perspectives: “dataset," “machine learning model," "cross-validation method," “sample size," and “publication year." Significant differences were found from the five perspectives (Table 1). Specifically, the *I*^2^ of the ADNI dataset (77%) was significantly larger than the other datasets (56%) (*P* = 0.032). This indicated that the “dataset" could be one of the potential factors of high heterogeneity. For the diagnostic model, the *I*^2^ of ML, DL, and others method were 74% (*P* < 0.001), 65% (*P* < 0.001), and <1% (*P* = 0.650), separately. Similarly, low heterogeneity existed among the studies with other cross-validation methods (*I*^2^ < 1%, *P* = 0.781). Studies with samples <150 showed lower heterogeneity in comparison to studies with large samples. Specifically, the *I*^2^ was 68% (samples 150–300), 68% (samples 300–450), and 78% (samples > 450). As for the publication year, the *I*^2^ of 2011–2014, 2015–2018, and 2019–2021 was higher than 65%, while the *I*^2^ of 2006–2010 was relatively lower. This is probably because few studies were included for 2006–2010. Similar patterns were obtained even within the ADNI dataset (Table 1). As shown in Fig. 4a, meta-regression analysis revealed a linear relationship between sample size and log (DOR) (*P* < 0.001), indicating that the sample size could be one of the potential factors of high heterogeneity. Furthermore, datasets, models, and cross-validation methods showed similar linear relationships with log(DOR) (all *P* < 0.05).

**Table 1:** Summary of heterogeneity in different subgroups based on a random effect model.

Subgroup	Type	ADNI + other datasets			ADNI		
		Number	Heterogeneity (*I*^2^ %)	*P* values	Number	Heterogeneity (*I*^2^ %)	*P* values
**Dataset**	ADNI	79	77 (*P* < 0.001)	*P* = 0.032			
	other datasets	37	56 (*P* < 0.001)				
**Method**	machine learning	70	74 (*P* < 0.001)	*P* < 0.001	48	79 (*P* < 0.001)	*P* = 0.002
	deep learning	30	65 (*P* < 0.001)		24	69 (*P* < 0.001)	
	other method	16	<1 (*P* = 0.650)		7	<1 (*P* = 0.620)	
**Validation**	*k*-fold	72	76 (*P* < 0.001)	*P* = 0.003	59	76 (*P* < 0.001)	*P* < 0.019
	leave-one-out	24	47 (*P* < 0.001)		11	60 (*P* < 0.001)	
	independent	15	80 (*P* < 0.001)		8	87 (*P* < 0.001)	
	other validation	5	<1 (*P* = 0.780)		1	Not applicable	
**Sample**	0–150	41	44 (*P* = 0.010)	*P* = 0.008	19	41 (*P* = 0.008)	*P* = 0.025
	150–300	32	73 (*P* < 0.001)		24	70 (*P* < 0.001)	
	300–450	30	74 (*P* < 0.001)		28	76 (*P* < 0.001)	
	>450	13	88 (*P* < 0.001)		8	92 (*P* < 0.001)	
**Year**	2006–2010	6	<1 (*P* = 0.510)	*P* < 0.001	2	8 (*P* = 0.300)	*P* < 0.003
	2011–2014	26	68 (*P* < 0.001)		14	65 (*P* < 0.001)	
	2015–2018	44	68 (*P* < 0.001)		34	74 (*P* < 0.001)	
	2019–2021	37	78 (*P* < 0.001)		29	81 (*P* < 0.001)	
**Total heterogeneity**			73 (*P* < 0.001)			77 (*P* < 0.001)	

### Analysis sensitivity

We performed sensitivity analysis is to explore whether the results were stable or not. The Baujat plot has been proposed to detect sources of heterogeneity in the meta-analysis (Anzures-Cabrera & Higgins, 2010; Baujat *et al*., 2002). As shown in Fig. 4b, studies that fell in the upper right corner of the graph made an enormous contribution to the heterogeneity. Note that the heterogeneity was still high ( *I*^2^ =  68%) after removing those studies, and the *I*^2^ was more prominent (i.e. larger than 71%) when we dropped any of the included studies. To sum up, the high heterogeneity existing among studies was included in our analysis.

### Reporting bias

**Figure 5:**
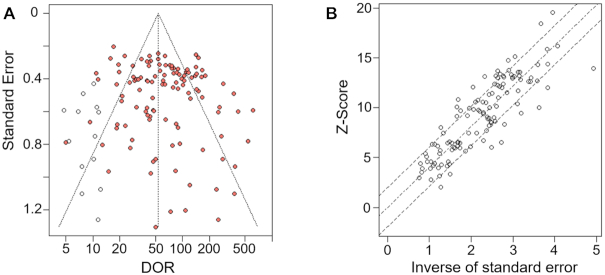
(A) Funnel chart of the trim and fill method. The red circles represent the original studies, and the hollow circles represent the studies added after cutting. (B) A radial plot of Egger's test. The dotted line in the middle represents the regression fitting line based on the random-effects model. The dashed lines on both sides represent the 95% confidence level.

As shown in Fig. 5a, the funnel chart was approximately symmetrical, indicating that there was no significant publication bias in the included studies. A large number of studies fell outside the dotted line (95% CI), which was associated with the heterogeneity among the included studies. Both *P* values of Begg's test (*P* = 0.367) and Egger's test (*P* = 0.068) were >0.05, which means that we could not reject our null hypothesis that there was no publication bias of the included studies in our analysis (Fig. 5b).

## Discussion

In this paper, we systematically reviewed 101 articles relevant to diagnostic models for AD and found high heterogeneity among them. Our meta-analysis showed that many factors contribute to the heterogeneity of high diagnostic accuracy of AD using sMRI, which included but was not limited to the "dataset," "machine learning model," "cross-validation method," "sample size," and "publication time". These complicated variables probably are reasons for hindering the clinical translation for the early diagnosis of AD.

Structural MRI is one of the most widely used and accessible techniques in searching the imaging biomarkers for AD and has been recommended in clinical guidelines (National Institute for Health and Care Excellence (UK), 2018). Modern machine learning models have been pervasive in searching neuroimaging-based biomarkers for AD based on sMRI, especially with the enormous contribution from the ADNI (http://adni.loni.usc.edu/) and other open datasets (https://www.oasis-brains.org). As mentioned previously, promising results have been achieved for classifying AD from NC with different models using sMRI (Cheng *et al*., 2017; Feng *et al*., 2021; Rathore *et al*., 2017). In the past decades, a lot of work has been done to translate the machine learning model from bench to bedside. Serval CAD tools have already been commercially available for diagnosing AD, such as Neuroquant (https://www.cortechslabs.com/neuroquant), cNeuro (https://www.combinostics.com/), mdbrain (https://mediaire.de/en/about/), icobrain dm (https://icometrix.com/products/icobrain-dm), and BIOMETRICA ( https://jung-diagnostics.de/de/diagnostics). However, these toolkits only can assess the volume of specific brain regions or some other brain features, which needs to be further evaluated by the radiologists. Therefore, there is still a gap from scientific research to clinical radiology practice.

First, the lack of generalizability and validation of large sample size is considered to be a major challenge for the field. Based on the included studies, we observed that sample size has been increasing quickly in recent years. We also noted that most of these studies are based on ADNI. Although the sample size of ADNI is >2000 participants, it comes from around 70 sites and four stages (ADNI1, ADNIGO, ADNI2, and ADNI3). It should be noted that the ADNI website (http://adni.loni.usc.edu/) is not a "click & play" curated dataset but a set of MRI scans and spreadsheets that need to be selected, downloaded, and processed by the individual researchers. Depending on the selection criteria, specific diagnostic groups are included. For example, the specific "early MCI" groups and newer ADNI3 patients show less (hippocampus) atrophy compared to the "late MCI" (Moore *et al*., 2020). In a word, it is still a significant heterogeneous dataset. The small sample size with leave-one-out or *k*-folds cross-validation will lead to the overfitting and/or the out-of-distribution problem for imaging data from different sites with various co-factors. Besides, the accuracy of the deep learning model is higher than the traditional machine learning model. One of the potential reasons is that the complex parameter space provides the possibility of higher accuracy; meanwhile, it also presents a challenge for generalization. Specifically, several recent studies with hippocampus markers (Ding *et al*., 2021; Li *et al*., 2019; Zhao *et al*., 2020) or deep learning models (Dyrba *et al*., 2021; Jin *et al*., 2020; Lian *et al*., 2020; Qiu *et al*., 2020) have provided a promising performance for introducing independent cross-validation with large multisite individuals. We believe that this kind of cross-validation with the independent sites would accelerate clinical translation.

Second, the lack of standardized preprocessing has hampered clinical application. As we know, the measures of the brain are also impacted by several technical factors such as the reliability and robustness of MRI scanners, as well as postprocessing pipelines. There are several popular tools, such as FSL, Freesurfer, Cat12, SPM, etc. However, we know that even the simplest measure, such as gray matter volume of the brain, would have minor differences driven by the used tools (Guo *et al*., 2019; Han *et al*., 2006; Jovicich *et al*., 2009; Medawar *et al*., 2021), which might affect the homogeneity of the models. It is impossible to ask all these studies to be performed at one single site, especially for large cohort studies. Therefore, a standard, well-established pipeline for data preprocessing is encouraged for future studies (Esteban *et al*., 2019; Glasser *et al*., 2013).

Third, some of the previous results were overestimated in the clinical availability due to over-selected participants who were involved in their studies. For example, the ADNI dataset is highly preselected in terms of image acquisition and patients via the rigorous inclusion and exclusion criteria. However, for a patient with cognitive impairment who decides to visit their doctor, he/she possibly has other disorders, such as diabetes, head trauma, hypertension, cerebrovascular disease, etc., and these risks would draw influence on the CAD system of AD. Many factors should be responsible for the significant heterogeneity rather than a single factor, and the heterogeneity of AD symptoms itself also seriously challenges and will prevent highly accurate prediction linking brain features for the development of a robust CAD system of AD (Ferreira *et al*., 2020; Habes *et al*., 2020; Machado *et al*., 2020; B. Zhang *et al*., 2021). Thus, a large, high-quality longitude protocol, validated in independent datasets obtained from different centers would benefit the future CAD system for AD.

This study has limitations. We only focused on the sMRI studies due to their wide availability as clinical tools. We tried to find the source of heterogeneity and evaluate the study's reliability. Given the high heterogeneity among studies in the current analysis, we focused on meta-regression, subgroup comparison, sensitivity analysis, and the evaluation of publication bias. Even so, there was still high heterogeneity for most subgroups. We speculate that adding other measures such as PET imaging, CSF markers, genetic factors, or lifestyle factors might help to achieve higher predictive power with lower heterogeneity.

## Conclusion

In summary, we found significant differences among the different diagnostic models, i.e., high heterogeneity, by systematically reviewing the diagnostic models for AD. This was due to many factors, including but not limited to the "dataset", "machine learning model", "cross-validation method", "sample size" and "publication time". It is a tremendous challenge for model promotion and clinical translation. The clinical translation of AD diagnostic models still has a long way to improve.
